# Left Atrial Appendage Occlusion Compared to Anticoagulation in Patients Suffering from Atrial Fibrillation with Advanced Chronic Kidney Disease

**DOI:** 10.3390/jcm14165709

**Published:** 2025-08-12

**Authors:** Sergio López-Tejero, Pablo Antúnez-Muiños, Pilar Fraile-Gómez, Fabián Blanco-Fernández, Gilles Barreira-de Sousa, Jesús Herrero-Garibi, Javier Rodríguez-Collado, Alejandro Diego-Nieto, Candelas Pérez del Villar, Gonzalo C. Delgado-Lapeira, Javier Martín-Moreiras, Pedro L. Sánchez-Fernández, Ignacio Cruz-González

**Affiliations:** 1 Servicio de Cardiología, Complejo Asistencial Universitario de Salamanca (CAUSA), Universidad de Salamanca (USAL), 37007 Salamanca, Spain; 2 Instituto de Investigación Biomédica de Salamanca (IBSAL), 37007 Salamanca, Spain; 3 Centro de Investigación Biomédica en Red de Enfermedades Cardiovasculares (CIBER-CV), Spain; 4 Servicio de Nefrología, Complejo Asistencial Universitario de Salamanca (CAUSA), 37007 Salamanca, Spain

**Keywords:** advanced chronic kidney disease, atrial fibrillation, anticoagulation, left atrial appendage occlusion

## Abstract

**Background/Objectives**: Chronic kidney disease (CKD) is a significant risk factor for thrombogenic and bleeding events in patients with atrial fibrillation (AF). Left atrial appendage occlusion (LAAO) is increasingly utilized as an alternative to oral anticoagulation. We aimed to compare LAAO against medical therapy in advanced CKD patients (A-CKD). **Methods**: We conducted a retrospective cohort study to compare patients with AF who had undergone LAAO (intervention group) or patients receiving oral anticoagulation (OAC) (control group). All of them had the diagnosis of A-CKD (estimated glomerular filtration rate (eGFR) < 30 mL/min/1.73 m^2^). The primary endpoint was a composite of stroke, transient ischemic attack (TIA), systemic embolism (SE), and major bleeding. Secondary endpoints included: an efficacy combined endpoint (a composition of stroke, TIA, and SE); major bleedings (defined as Bleeding Academic Research Consortium (BARC) ≥ 3), and mortality at follow-up. A propensity score matching was used to balance the populations. **Results**: In total, 81 and 102 patients composed the LAAO and anticoagulation groups. Mean age was 78.27 ± 10.3 and 81.2 ± 9.07 (*p* = 0.069) and female sex was 38.3% and 44.1%, respectively. Patients who underwent LAAO had a higher HAS-BLED score: 3.46 ± 0.85 vs. 3.77 ± 1.06, *p* = 0.011. Median follow-up was 19.0 months [IQR: 10.9–33.5]. There were no differences in the primary combined endpoint at 3-years follow-up—22.2% vs. 34.2% (hazard ratio (HR) 0.63, CI-95%: 0.353–1.11, *p* = 0.102)—nor respecting the efficacy combined endpoint: 3.7% vs. 6.9% (HR 0.54, CI-95%: 0.14–2.09, *p* = 0.355). Patients under anticoagulation treatment did present major bleedings (BARC ≥ 3) more often than the intervention group: 38.3%vs50% (HR 0.52, CI-95%: 0.28–0.96, *p* = 0.031). A total of 15 patients (14.7%) from the control group underwent LAAO during follow-up. After a propensity score matching analysis, the primary combined endpoint was more frequent in the control group (HR 0.47, CI-95%: 0.25–0.90, *p* = 0.019). **Conclusions**: Compared with oral anticoagulation therapy, LAAO had no differences in efficacy, but fewer major bleeding rates were found.

## 1. Introduction

AF is the most common sustained cardiac arrhythmia in adults, with an estimated prevalence of 2–4% [1]. Stroke is the most debilitating complication of AF [2]. CKD is a frequent comorbidity in AF patients, whose global prevalence is estimated at over 13.4% [3]. For those patients suffering from CKD and a high thromboembolic risk, there is a paradoxical rise in bleeding events [4]. Each 10 mL/min/1.73 m^2^ eGFR reduction is associated with a 9% rise in hemorrhagic risk [5]. Furthermore, there is a lack of evidence of the safety and efficacy of direct oral anticoagulants (DOACs) in patients with A-CKD, (eGFR < 30 mL/min/1.73 m^2^), as these patients were systematically excluded from the pivotal trials: RE-LY, ROCKET-AF, ARISTOTLE, and ENGAGE-AF-TIMI 48 [6,7,8,9]. In addition, the role of warfarin in the advanced CKD cohort is also unclear. For example, a meta-analysis conducted by Randhawa et al. reported that there were no differences in stroke, embolism, or death with an increasing risk of hemorrhagic stroke in end-stage renal disease patients receiving warfarin [10]. Recent data from the Swedish National Cohort showed a lower bleeding rate with no differences in the stroke rate for DOACs compared to warfarin and a possible increase in mortality [11]. Recently, two randomized trials have been published comparing apixaban versus vitamin K antagonists for patients undergoing hemodialysis. In the AXADIA-AFNET 8 Study, apixaban seems to be safer than phenprocoumon, although there was a global high rate of thrombotic events in both groups, despite the anticoagulation [12]. In the RENAL-AF trial, despite being stopped prematurely, the bleeding rate was superior to 25%, and apixaban may have a higher likelihood of death, although the study was underpowered [13].

LAAO is a percutaneous technique which has been shown to reduce the requirement of anticoagulation therapy in non-valvular AF patients, being non-inferior to oral anticoagulation in stroke prevention, with a bleeding rate reduction in randomized trials [14,15]. Around 90% of thrombi in the left atrium are originated in the left atrium appendage [16,17], this percentage is even higher in CKD patients, due to its influence on the prothrombotic state [18]. Therefore, LAAO could be safe and effective in A-CKD patients.

The aim of this study was to assess the efficacy and safety of LAAO at follow-up in patients with severe renal impairment and compare its results with a similar cohort of A-CKD patients under medical treatment. To the best of our knowledge, this is the first study that compares LAAO versus OAC in an A-CKD population, including a propensity score matching analysis.

## 2. Methods

This study represents a retrospective analysis of patients suffering from AF with advanced chronic kidney disease. Two populations were included: the LAAO group (intervention) and the anticoagulation group (control). The intervention group was composed of patients with AF who suffered from A-CKD (defined as eGFR < 30 mL/min/1.73 m^2^, G4 and G5, or patients receiving dialysis, G5D) and underwent LAAO from December 2009 to May 2022 in our center.

**Figure 1:**
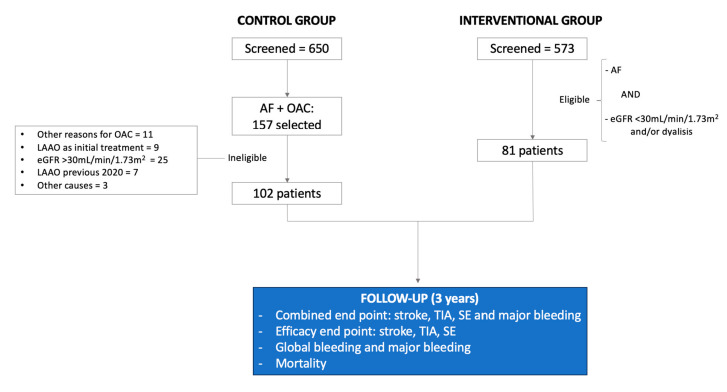
Study design.

The control group was selected from those patients under follow-up at the chronic kidney disease outpatient clinics supervised by the Nephrology Department from our center in 2020. The eligibility criteria for this group were (1) AF requiring anticoagulation and (2) A-CKD defined as eGFR < 30 mL/min/1.73 m^2^ or undergoing hemodialysis (Figure 1). All eligible patients met this criteria. Patients requiring anticoagulation for other causes (such as mechanical prosthesis, deep vein thrombosis, etc.) were excluded. Follow-up was carried out using any medical records until 30 June 2023. Patients from the control group who underwent LAAO during follow-up were also analyzed (cross-over), and follow-up was stopped at the time they received the intervention.

All clinical data, in-hospital, and follow-up outcomes were prespecified in the online database used by our center, complying with the Law on Data Protection requirements, and were accessible only to participating operators and registry coordinators. The study protocol adhered to the 1975 Helsinki Declaration for Ethical Treatment of Human Subjects, with local ethics committee approval (Código CEIm PI 2023-05-1291).

### 2.1. Study Endpoints

The primary endpoint was a composite of stroke, TIA, SE, and major bleeding. Secondary endpoints included an efficacy combined endpoint (a composition of stroke, TIA, and SE), the rate of major bleeding, and mortality at follow-up. Stroke, TIA, and SE were defined as any medical encounter with any of them as a final clinical diagnosis. Relevant bleeding during hospitalization was defined as any bleeding which leads to blood transfusion. Major bleeding during follow-up was defined as BARC ≥ 3.

### 2.2. Statistical Analysis

For categorial variables, the frequency of each category is presented, and comparison between groups was performed using the chi-square test. For continuous variables, the Shapiro–Wilk test was used to identify if the distribution was normal, and is shown as a mean (±standard deviation) in that case or as a median [interquartile range (IQR)] otherwise. Comparisons between groups were performed using the T-test or Mann–Whitney U test according to the distribution of the variables. The incidence of ischemic and bleeding complications was recorded. The relative risk reduction (RRR) was calculated using the adjusted stroke or bleeding rate per year predicted by scores (CHA_2_DS_2_-VASc and HASBLED, respectively) and the observed rate in our cohorts for each treatment group. All the study endpoints at 1 and 3 years were analyzed by Kaplan–Meier event-free survival curves, and comparisons were performed using the log-rank test. They were expressed as a probability rate displayed on the curves and the range goes from 0 and 1. Hazard ratios (HRs) with 95% confidence intervals were obtained by using the Cox model for survival comparison between groups. All tests were 2-sided at the 0.05 significance level.

Propensity score matching was used to balance the confounding factors in each treatment group. The propensity score was calculated using a logistic regression between groups based on treatment and depending on 4 characteristics: age, gender, very high thromboembolic risk (CHA_2_DS_2_-VASc ≥ 4), and bleeding risk (HASBLED ≥ 3). Patients were matched 1:1 following these characteristics and a propensity score using a caliper <0.2. We found 71 pairs of patients with similar characteristics and no differences.

Categorical analysis and group comparisons were performed using Jamovi version 2.3.21.0. Survival distribution following Kaplan–Meier method, propensity score matching, and its results were performed using Stata V15.

## 3. Results

A total of 573 patients who underwent LAAO were analyzed. A total of 81 patients (14%) had been diagnosed with A-CKD before the intervention. On the other hand, from the 650 patients followed by Nephrology during 2020, we identified 157 who had AF. Finally, 102 patients defined the control group since 55 were excluded because they did not fulfill the eligibility criteria (Figure 1).

### 3.1. Baseline Characteristics of the Study Population

Table 1 includes the main baseline characteristics of the populations analyzed. Demographics were similar between groups, with differences found in age, prior stroke, patients receiving hemodialysis, prior bleeding, and the different types of bleeding. There were no differences between sex (female in control group 44.1% vs. intervention group 38.3%, *p* = 0.425) and cardiovascular risk factors. Prior stroke (5.9% vs. 18.5%, *p* = 0.008) and prior bleeding (28.4 vs. 80.2%, *p* < 0.001) were more frequent in the intervention group. The CHA_2_DS_2_-VASc score was similar between groups (4.57 ± 1.39 vs. 4.7 ± 1.44, *p* = 0.603), although control group patients had a lower bleeding risk according to the HAS-BLED score (3.46 ± 0.85 vs. 3.77 ± 1.06, *p* = 0.011).

Creatinine levels and renal function were similar (3.49 ± 1.9 mg/dL vs. 3.9 ± 2.15 mg/dL, *p* = 0.168) (eGFR: 17.4 ± 7.0 mL/min/1.73 m^2^ vs. 16.9 ± 8.26 mL/min/1.73 m^2^, *p* = 0.640). The CKD stages were as follows: stage 4 64.7% (control group) vs. 51.9% (intervention group); stage 5 10.8% vs. 7.4%, and hemodialysis 24.5% vs. 40.7% (*p* = 0.062). There was a significant difference found for individuals undergoing hemodialysis between groups (24.5% control vs. 40.7% intervention, *p* = 0.019).

### 3.2. Procedural Characteristics and In-Hospital Results

The main indication for LAAO was prior bleeding (74.1%), followed by a high risk of bleeding without any episodes (11.1%). Watchman (48.1%) and Amulet (32.1%) were the most common devices, achieving 100% procedural success. LAAO intervention was guided by transesophageal echocardiography (TEE) in 94.9%, whereas the rest was by intracardiac echocardiography (ICE). There were only two intraprocedural (2.5%) complications: one cardiac tamponade managed with pericardial drainage and one vascular complication. No death was associated with the procedure. In-hospital follow-up showed four relevant bleedings (4.9%) and one death from a respiratory etiology in a patient with previous severe respiratory disease. No strokes were recorded.

### 3.3. Follow-Up Outcomes

**Figure 2:**
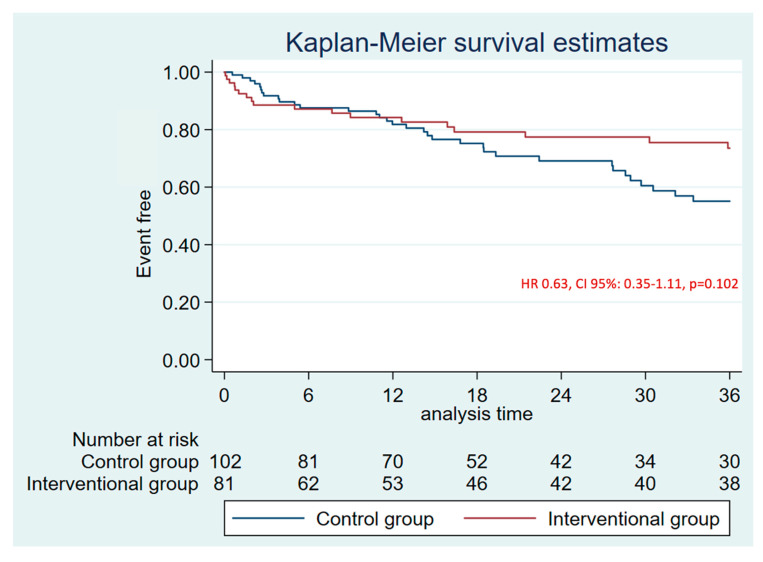
3-years follow-up Kaplan–Meier plot and estimate for primary combined endpoint (stroke, AIT, SE, and major bleeding).

**Figure 3:**
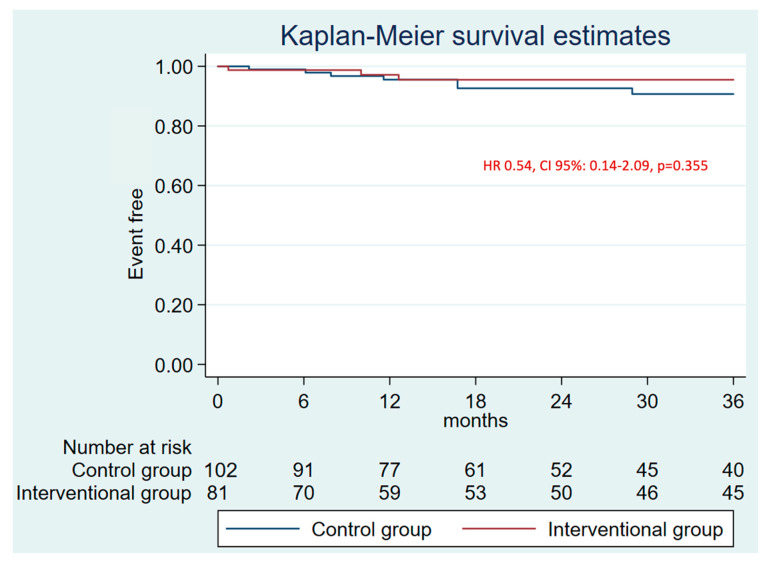
3-years follow-up Kaplan–Meier plot and estimate for the efficacy endpoint (stroke, AIT, and SE).

**Figure 4:**
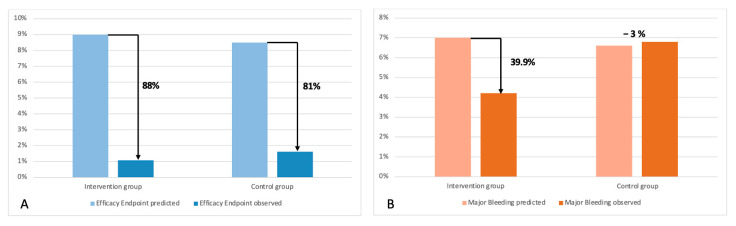
Efficacy endpoint RRR (**A**). Major bleeding RRR (**B**).

The median follow-up was 19.0 months [IQR: 10.9–33.5]. There were no differences in the primary combined endpoint at 1 year (HR 0.94, CI 95%: 0.45–1.98, *p* = 0.879) (Figure S1) and at 3 years (HR 0.63, CI 95%: 0.353–1.11, *p* = 0.102) (Figure 2, Table 2). No differences were found in the efficacy combined endpoint either (Figure 3). The annual rate for the efficacy endpoint was 1.07 for the patients who underwent LAAO and 1.62 per 100 patients/year for the control group. These rates explain a higher RRR for patients who underwent LAAO (88% vs. 81%) (Figure 4A).

Differences in treatment were found during follow-up (*p* < 0.001). Most of the patients who underwent LAAO received SAPT (55%) and DAPT (21.25%) at 6-months follow-up. Moreover, there was a significant percentage of them (16.5%) who did not receive any antithrombotic therapy. Only three patients had device-related thrombosis (two during the first 6 months and one between the sixth and the twelfth month). Two of them remained with DAPT, and one was treated with warfarin. On the other hand, a large proportion of the control patients were receiving anticoagulation treatment (warfarin 55% and DOAC 23%).

**Figure 5:**
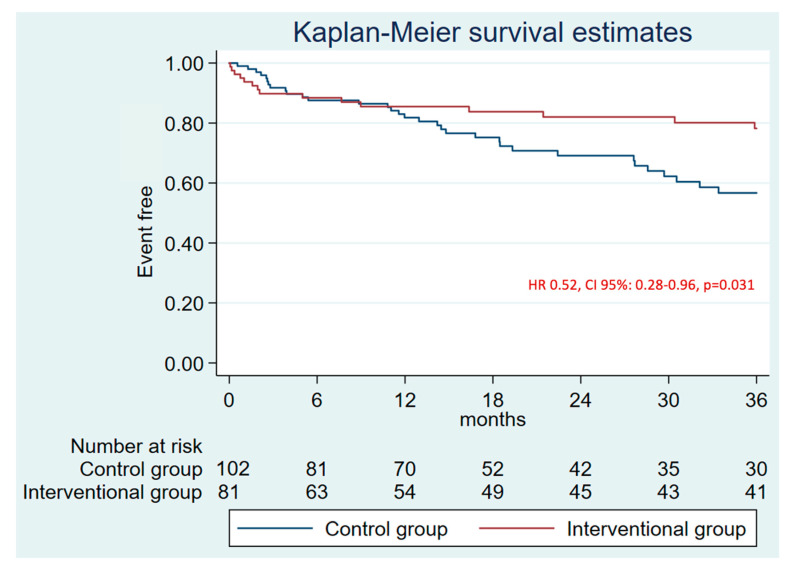
3 years follow-up Kaplan-Meier plot and estimate for major bleeding.

**Figure 6:**
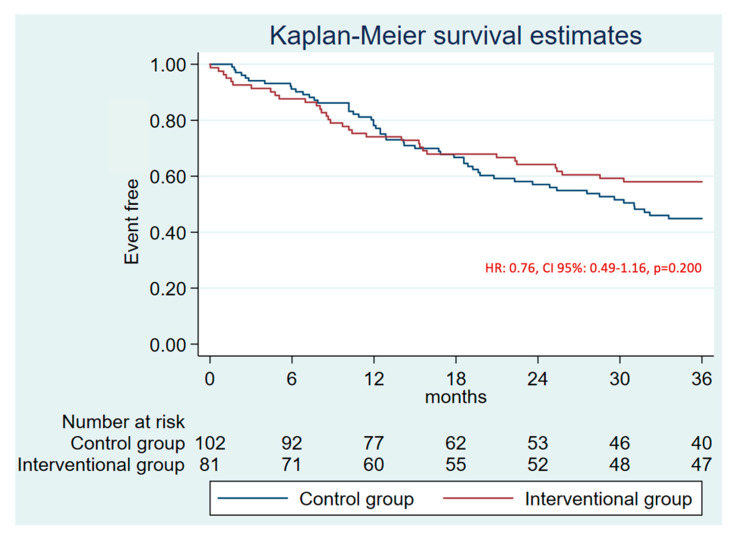
3 years follow-up Kaplan-Meier plot and estimate for mortality.

There were no differences in major bleeding (defined as BARC ≥ 3) at 1 year between groups (HR 0.86, CI 95%: 0.40–1.83, *p* = 0.685) (Figure S2). However, there was a lower major bleeding rate in the intervention group at 3-years follow-up (HR 0.52, CI 95%: 0.28–0.96, *p* = 0.031) (Figure 5). The annual rate was 4.21 and 6.8 per 100 patients/year for the intervention and control group, respectively. Hence, we found a 39.9% RRR for patients after LAAO, whereas an increased risk of 3% was observed for patients who received anticoagulation (Figure 4B). Finally, mortality was similar between both groups at 3 years (HR: 0.76, CI 95%: 0.49–1.16, *p* = 0.200) (Figure 6).

In total, 15 patients (14.7%) from the control group underwent LAAO at follow-up (cross-over). The reason leading the intervention was bleeding for all patients except one. The median follow-up until cross-over was 12.4 months [IQR: 10.1–16.7]. The success in this cross-over group was 100%. For those patients, there were no procedural-related or in-hospital complications, and no death was registered. During follow-up, one patient had a stroke during a transcatheter aortic valve replacement intervention, and one had an intestinal infarction despite no DRT being observed. No major bleedings were recorded.

**Figure 7:**
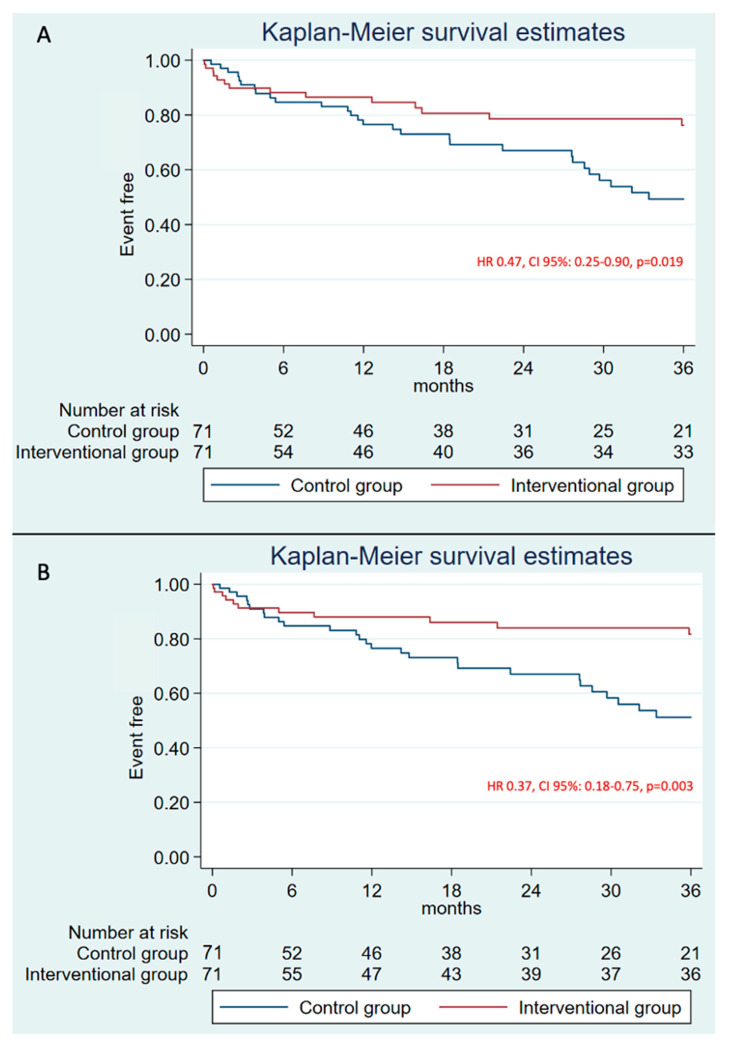
3-years follow-up Kaplan–Meier plot after propensity score matching. They estimate for the primary combined endpoint (**A**) and major bleeding (**B**).

After propensity score analysis, we identified 71 equal pairs of patients. Table 3 summarizes the baseline population characteristics after the propensity score matching. No differences could be appreciated. They were also similar according to very high risk of stroke (*p* = 0.32) and high risk of bleeding (*p* = 0.44). Similar outcomes were shown in the primary combined endpoint at 1-year follow-up (HR 0.62, CI 95%: 0.27–1.41, *p* = 0.246). Otherwise, it was found less frequently in the intervention group at 3 years (HR 0.47, CI 95%: 0.25–0.90, *p* = 0.019) (Figure 7A). In fact, major bleeding was also more frequent in the control group compared to the intervention group at 3 years (HR 0.37, CI 95%: 0.18–0.75, *p* = 0.003) (Figure 7B). Mortality remained similar between groups (HR 0.96, CI 95%: 0.59–1.57, *p* = 0.879) (Figure S3).

## 4. Discussion

The main findings of our study were (a) LAAO is an effective and safe treatment for stroke prevention in A-CKD patients, comparable to standard therapy, and (b) major bleeding events were lower in LAAO group compared with the medical treatment group.

Our study aimed to compare LAAO with standard treatment (anticoagulation) in A-CKD patients, which is a cohort that has been excluded from major trials for DOACs [6,7,8,9]. LAAO results in patients with renal impairment have already been published. Faroux et al. reported good results with no differences in all periprocedural complications and no increased risk of ischemic stroke (HR 0.65, CI 95%: 0.22–1.92; *p* = 0.435) at 2-year follow-up in patients with eGRF < 45 mL/min/1.73 m^2^ [19]. Along the same lines, our group has published similar outcomes for stroke and major bleeding prevention in A-CKD patients undergoing LAAO compared to those without such renal conditions [20]. However, a lack of evidence is still present for A-CKD patients. In the present study, procedural success was 100%. The technical success rate has been rising since it was 91% in PROTECT-AF [14], increasing to 96.8% in PRAGUE-17 [21], and, recently, the National Cardiovascular Data Registry (NCDR) from the USA reported a success rate of 98.3% [22]. Only two intraprocedural complications were registered in our study: one vascular complication and one cardiac tamponade, managed with pericardial drainage. There was only one in-hospital death, but it was unrelated to the procedure (severe respiratory insufficiency due to advanced chronic pulmonary disease). The technical success and absence of complications in cross-over patients are remarkable. These results emphasize the safety of LAAO.

There were no differences in the primary combined endpoint between both groups. The Prague-17 trial also evaluated a combined endpoint, as ours did; they included death and device-related complications too, with no differences found (HR 0.81, CI 95%: 0.56–1.18, *p* = 0.27) [23]. However, in our study, after the propensity score matching analysis, differences were achieved in the primary combined endpoint at 3 years. Therefore, LAAO could potentially be considered an alternative treatment of choice for A-CKD patients compared to the standard anticoagulation therapy. In addition, there was a low rate of ischemic events in the LAAO group (1.9% annual rate), although it was slightly higher than the one reported by Faroux et al. (1.6%) but lower than Kefer et al. for the renal population (2.3%) [19,24]. Concerning DOAC, the apixaban ischemic events rate has been 2.81%, which is lower than rates seen on warfarin (5.06%) in patients with eGFR 25–30 mL/min/1.73 m^2^ [25]. Even so, their rate of ischemic events was higher than our sample. Recently, data from the Swedish National Registry have reported stroke rates as high as 2.2% in anticoagulated patients suffering from A-CKD [11]. Furthermore, Genovesi et al. found a lower rate of embolic events in patients requiring dialysis who underwent LAAO against those anticoagulated (0% vs. 3.9%, *p* = 0.092) or without any treatment (0% vs. 8%, *p* = 0.021) [26]. Indeed, our annual rate was lower than expected according to CHA_2_DS_2_-VASc for the combined ischemic endpoint [27], which implies a relative reduction risk of 88% and 80%, respectively, with respect to the adjusted stroke or bleeding rate per year. This would suggest additional benefits from LAAO for the prevention of ischemic events. This trend has been also reported in each CKD stage [28]. Furthermore, it is important to note that the current literature is yet to demonstrate evidence that OAC in A-CKD patients reduces the ischemic events compared with no therapy, particularly in patients on renal replacement therapy (RRT). This highlights the important gap in evidence that our study addresses and the utility of LAAO in the under-represented population of individuals with A-CKD and atrial fibrillation.

Most LAAO studies are focused on major/relevant bleedings. Despite not being our primary endpoint, the major bleeding outcomes became the cornerstone of our findings. Evidence from observational studies showed that DOACs in patients with CKD have reduced global and major bleeding [11,25], GI bleeding [28], or intracranial bleeding [10] compared to warfarin. However, the latest results from randomized controlled trials do not support these findings in dialysis patients [12,13]. It is well known that patients suffering from CKD have a higher bleeding risk, especially when they are receiving antithrombotic treatment [29]. In our study, at 1-year follow-up, there were no differences in major bleeding between groups (HR 0.86, CI 95%: 0.40–1.83, *p* = 0.685), although there was at 3 years (HR 0.52, CI 95%: 0.28–0.96, *p* = 0.0307). This may be related to the antiplatelet treatment after LAAO during the initial months. The ADALA study—pending publication—suggests a higher rate of bleeding due to DAPT compared to low doses of anticoagulation. Another reason could be that hemorrhages were the main indication for LAAO, and a previous hemorrhage in individuals has been shown to be the main predictor for further bleeding events [30,31]. Interestingly, Hildick-Smith et al. also published a similar pattern in non-renal populations after LAAO [32]. Similarly, a study focused on dialysis patients in Italy showed differences between groups at long-term follow-up (HR 6.48, CI 95%: 1.32–31.72, *p* = 0.021) [26]. The annual bleeding rate for the LAAO group (4.21 per 100 patients/year) was higher than in Prague-17 at 4-years follow-up, which can be explained because these patients had a lower risk of bleeding (HASBLED 3.1 ± 0.9 vs. 3.77 ± 1.06) added to their renal condition too. Luani et al. also reported a lower bleeding rate in CKD patients [33]. Nevertheless, data from real-life renal registries showed higher rates of major bleeding than those reported in this study: 9.8 per 100 patients/year by Faroux et al. [19] and 11.7 per 100/patients/year by Benini-Tapias et al. [34]. On the other hand, anticoagulation also presents a risk of bleeding in A-CKD patients. Rivaroxaban achieved 8.44 per 100 patients/year bleeding events in patients with A-CKD, while warfarin had 9.39 [35]. Welander et al. analyzed this trend, finding a rate of bleeding of 5.6% and 17.4 for DOAC therapy [11]. The increasing risk of bleeding in patients who underwent anticoagulation (RRR -3) in our population must be highlighted, which follows the evidence suggesting that anticoagulation may be harmful in terms of bleeding for CKD patients [36,37,38]. The protective role of LAAO in CKD, including a clear RRR in each stage, has been reported [28]. After the propensity score matching, differences between groups expanded, which reinforces the hypothesis that LAAO could be an attractive alternative to OAC in A-CKD patients. The ongoing LAA-KIDNEY trial (NCT05204212) will provide more accurate information about this treatment in dialysis patients.

Propensity score matching was performed to balance the population. The resulting cohorts were equal in terms of baseline characteristics. Due to their comorbidities, all the patients had a high risk of embolism (CHA_2_DS_2_-VASc ≥ 2); therefore, a very high risk of a thromboembolic event (CHA_2_DS_2_-VASc ≥ 4) was implemented. Melgaard et al. found the absolute risk of ischemic stroke, thromboembolism, and death was raised for those patients with HF and a CHA_2_DS_2_-VASc ≥ 4 [39]. This risk was reproducible even in patients without AF. It was confirmed in a recent meta-analysis encompassing more than 45.000 patients, showing an increased susceptibility to stroke [40]. Similar to this issue, HASBLED was adjusted for patients who had a high risk (HASBLED ≥ 3) [41]. Despite both groups being similar after the matching, larger differences were observed in the primary combined endpoint and in major bleedings, as was stated previously. Although this is a retrospective study, it does present important insight into a cohort with a paucity of literature and demonstrates in our cohort a high rate of procedural success and safety, with a signal to reduce bleeding without a difference in ischemic events in a high-risk population.

Finally, almost half of the patients had died at the 3-year follow-up. CKD is a predictor of adverse events and mortality [42], so since our population has high comorbidity associated with A-CKD, it explains this high death rate.

### Limitations

These analyses were based on observational and retrospective registry data with the inherent limitations of this study type. This nature and the limited size sample may lead to a lack of statistical power for achieving differences. The temporal gaps between both cohorts may introduce possible confounding, although the mortality for A-CKD patients has been stable over the last decades. Despite statistical adjustments, the effect of unmeasured confounding in the results cannot be ruled out. Therefore, the authors consider that it is reasonable to accept the positive results of LAAO in bleeding prevention. Treatment selection was chosen by the physician responsible. Although this study included the largest cohort of patients with A-CKD for one single center, this cohort is relatively small compared with the non-CKD group and therefore may limit the interpretation of findings. There was no core lab for event revision. In light of these limitations, the study findings may be considered as hypothesis-generating.

## 5. Conclusions

LAAO is an effective treatment option for ischemic and bleeding prevention when compared to medical treatment for patients with A-CKD and AF. In fact, there were lower major bleeding rates in comparison with anticoagulation. Further randomized studies are warranted to determine the final role of LAAO in A-CKD patients.

## Figures and Tables

**Table 1:** Clinical characteristics at baseline.

	Control Group *n* = 102	Intervention Group *n* = 81	*p*
Sex, female	45 (44.1)	31 (38.3)	0.425
Age	81.2 ± 9.07	78.2 ± 10.3	0.069
Hypertension	95 (93.1)	73 (90.1)	0.460
Dyslipidemia	69 (67.6)	47 (58)	0.241
Diabetes mellitus	45 (44.1)	42 (51.9)	0.298
Prior stroke	6 (5.9)	15 (18.5)	0.008 *
Prior hemorrhagic stroke	1 (1)	3 (3.7)	0.211
Prior TIA	4 (3.9)	3 (3.7)	0.939
Peripheral artery disease	17 (16.7)	16 (19.8)	0.590
Prior coronary artery disease	26 (25.5)	21 (25.9)	0.947
Prior PCI	13 (12.7)	14 (17.3)	0.390
Prior CABG	6 (5.9)	4 (4.9)	0.780
Prior heart failure	50 (49)	36 (44.4)	0.538
Prior cancer	27 (26.2)	21 (25.9)	0.934
Prior liver disease	4 (3.9)	5 (6.2)	0.484
Labile INR	14 (13.9)	19 (23.5)	0.095
Previous bleeding	29 (28.4)	65 (80.2)	<0.001 *
Prior relevant bleeding	17 (16.7)	58 (71.6)	<0.001 *
Prior intracranial bleeding	2 (2)	7 (8.6)	0.038 *
Prior GI bleeding	10 (9.8)	43 (53.1)	<0.001 *
CHA_2_DS_2_-VASc average ± DE	4.57 ± 1.39	4.7 ± 1.44	0.522
HASBLED average ± DE	3.46 ± 0.85	3.77 ± 1.06	0.033 *
Cr levels average ± DE	3.49 ± 1.9	3.9 ± 2.15	0.168
eGRF—CKDEPI average ± DE	17.4 ± 7.0	16.9 ± 8.26	0.640
LVEF (%) average ± DE	57.3 ± 12.4	57.0 ± 10.7	0.853
Type of AF 0.801			
Paroxysmal	33 (35.1)	27 (36)	
Persistent	16 (17)	10 (13.3)	
Permanent	45 (47.9)	38 (50.7)	
Baseline treatment			
No antithrombotic treatment	3 (2.94)	9 (11.1)	0.027 *
SAPT	6 (5.8)	8 (9.9)	0.313
DAPT	0	3 (3.7)	0.050 *
Warfarin	60 (58.8)	33 (40.7)	0.015 *
DOACs	22 (21.6)	13 (16.1)	0.346
Heparin	5 (4.9)	8 (9.9)	0.193
SAPT + anticoagulation	6 (5.9)	7 (8.6)	0.470

*: statistical significance. CABG: coronary artery bypass graft; Cr: creatinine; DAPT: dual antiplatelet therapy; DOAC: direct oral anticoagulation; eGFR: estimated glomerular filtration rate; GI: gastro-intestinal; INR: international normalized ratio; LVEF: left ventricle ejection fraction; PCI: percutaneous coronary intervention; SAPT: single antiplatelet therapy, TIA: transient ischemic attack.

**Table 2:** Follow-up outcomes.

	Control Group *n* = 102	Intervention Group *n* = 81	*p*
3-years FU stroke	5 (4.9)	1 (1.23)	0.166
3-years FU stroke + TIA + SE	7 (6.9)	3 (3.7)	0.35
3-years FU global bleeding	51 (50)	31 (38.3)	0.11
3-years FU major bleeding	36 (35.3)	18 (22.2)	0.05 *
3-years FU mortality	53 (51.9)	34 (41.9)	0.18
FU antithrombotic therapy 6 months <0.001			
No antithrombotics	4 (4)	13 (16.25)	
SAPT	8 (8)	44 (55)	
DAPT	1 (1)	17 (21.25)	
Warfarin	55 (55)	2 (2.5)	
DOAC	23 (23)	1 (1.25)	
Antiplatelet + anticoagulation	4 (4)	2 (2.5)	

*: statistical significance. DAPT: dual antiplatelet therapy; DOAC: direct oral anticoagulation; SAPT: single antiplatelet therapy; SE: systemic embolism; TIA: transient ischemic attack.

**Table 3:** Clinical characteristics at baseline after propensity score matching.

	Control Group *n* = 71	Intervention Group *n* = 71	*p*
Sex, female (%)	26 (37)	29 (41)	0.61
Age	79.1 ± 9.0	80.5 ± 7.8	0.309
Hypertension	66 (93.0)	63 (88.7)	0.38
Dyslipidemia	48 (67.6)	40 (56.3)	0.17
Diabetes mellitus	36 (50.7)	34 (47.9)	0.74
Peripheral artery disease	14 (19.7)	12 (16.9)	0.66
Prior coronary artery disease	19 (26.8)	16 (22.5)	0.56
Prior heart failure	35 (49.3)	30 (42.3)	0.40
Prior cancer	22 (31.0)	20 (28.2)	0.71
Prior liver disease	4 (5.6)	4 (5.6)	1.00
Labile INR	11 (15.5)	17 (23.9)	0.21
CHA_2_DS_2_-VASc (≥4)	52 (73.2)	57 (80.3)	0.32
HASBLED (≥3)	61 (85.9)	64 (90.1)	0.44

INR: international normalized ratio.

## Data Availability

The original contributions presented in this study are included in the article/Supplementary Material. Further inquiries can be directed to the corresponding author.

## Supplementary Materials

The following supporting information can be downloaded at: https://www.mdpi.com/article/10.3390/jcm14165709/s1, Figure S1: 1-year follow-up Kaplan-Meier plot and estimate for the primary combined endpoint (stroke, AIT, SE, and major bleeding) at 1-year follow-up; Figure S2: 1-year follow-up Kaplan-Meier plot and estimate for major bleeding; Figure S3: 3-year follow-up Kaplan-Meier plot and estimate for mortality after propensity score matching.

## Abbreviations

A-CKD	advanced chronic kidney disease
AF	atrial fibrillation
BARC	Bleeding Academic Research Consortium
DAPT	Dual Antiplatelet Therapy
DOAC	direct oral anticoagulant
eGFR	estimated glomerular filtration rate
GI	gastro-intestinal
HR	hazard ratio
ICE	intracardiac echocardiography
IQR	interquartile range
LAAO	left atrial appendage occlusion
OAC	oral anticoagulation
RRR	relative risk reduction
RRT	renal replacement therapy
SAPT	single antiplatelet therapy
SE	systemic embolism
TEE	transesophageal echocardiography
TIA	transient ischemic attack

## References

[1] Benjamin E.J., Muntner P., Alonso A., Bittencourt M.S., Callaway C.W., Carson A.P., Chamberlain A.M., Chang A.R., Chang S., Das S.R. (2019). Heart Disease and Stroke Statistics-2019 Update: A Report from the American Heart Association. Circulation.

[2] Alkhouli M., Noseworthy P.A., Rihal C.S., Holmes D.R. (2018). Stroke Prevention in Nonvalvular Atrial Fibrillation: A Stakeholder Perspective. J. Am. Coll. Cardiol..

[3] Hill N.R., Fatoba S.T., Oke J.L., Hirst J.A., O’Callaghan C.A., Lasserson D.S., Hobbs F.D. (2016). Global Prevalence of Chronic Kidney Disease—A Systematic Review and Meta-Analysis. PLoS ONE.

[4] Ding W.Y., Gupta D., Wong C.F., Lip G.Y.H. (2021). Pathophysiology of atrial fibrillation and chronic kidney disease. Cardiovasc. Res..

[5] Molnar A.O., Bota S.E., Garg A.X., Harel Z., Lam N., McArthur E., Nesrallah G., Perl J., Sood M.M. (2016). The risk of major hemorrhage with CKD. J. Am. Soc. Nephrol..

[6] Connolly S.J., Ezekowitz M.D., Yusuf S., Eikelboom J., Oldgren J., Parekh A., Pogue J., Reilly P.A., Themeles E., Varrone J. (2009). Dabigatran versus Warfarin in Patients with Atrial Fibrillation. N. Engl. J. Med..

[7] Patel M.R., Mahaffey K.W., Garg J., Pan G., Singer D.E., Hacke W., Breithardt G., Halperin J.L., Hankey G.J., Piccini J.P. (2011). Rivaroxaban versus Warfarin in Nonvalvular Atrial Fibrillation. N. Engl. J. Med..

[8] Granger C.B., Alexander J.H., McMurray J.J.V., Lopes R.D., Hylek E.M., Hanna M., Al-Khalidi H.R., Ansell J., Atar D., Ave-zum A. (2011). Apixaban versus Warfarin in Patients with Atrial Fibrillation. N. Engl. J. Med..

[9] Giugliano R.P., Ruff C.T., Braunwald E., Murphy S.A., Wiviott S.D., Halperin J.L., Waldo A.L., Ezekowitz M.D., Weitz J.I., Špinar J. (2013). Edoxaban versus Warfarin in Patients with Atrial Fibrillation. N. Engl. J. Med..

[10] Randhawa M.S., Vishwanath R., Rai M.P., Wang L., Randhawa A.K., Abela G., Dhar G. (2020). Association Between Use of Warfarin for Atrial Fibrillation and Outcomes Among Patients With End-Stage Renal Disease: A Systematic Review and Meta-analysis. JAMA Netw. Open..

[11] Welander F., Renlund H., Dimény E., Holmberg H., Själander A. (2023). Direct oral anticoagulants versus warfarin in patients with non-valvular atrial fibrillation and CKD G3-G5D. Clin. Kidney J..

[12] Reinecke H., Engelbertz C., Bauersachs R., Breithardt G., Echterhoff H.-H., Gerß J., Haeusler K.G., Hewing B., Hoyer J., Juergensmeyer S. (2023). A Randomized Controlled Trial Comparing Apixaban with the Vitamin K Antagonist Phenprocoumon in Patients on Chronic Hemodialysis: The AXADIA-AFNET 8 Study. Circulation.

[13] Pokorney S.D., Chertow G.M., Al-Khalidi H.R., Gallup D., Dignacco P., Mussina K., Bansal N., Gadegbeku C.A., Garcia D.A., Garonzik S. (2022). Apixaban for Patients with Atrial Fibrillation on Hemodialysis: A Multicenter Randomized Controlled Trial. Circulation.

[14] Holmes D.R., Reddy V.Y., Turi Z.G., Doshi S.K., Sievert H., Buchbinder M., Mullin C.M., Sick P. (2009). Percutaneous closure of the left atrial appendage versus warfarin therapy for prevention of stroke in patients with atrial fibrillation: A randomised non-inferiority trial. Lancet.

[15] Holmes DRJr Kar S., Price M.J., Whisenant B., Sievert H., Doshi S.K., Huber K., Reddy V.Y. (2014). Prospective randomized evaluation of the Watchman Left Atrial Appendage Closure device in patients with atrial fibrillation versus long-term warfarin therapy: The PREVAIL trial. J. Am. Coll. Cardiol..

[16] Blackshear J.L., Odell J.A. (1996). Appendage obliteration to reduce stroke in cardiac surgical patients with atrial fibrillation. Ann. Thorac. Surg..

[17] Pollick C., Taylor D. (1991). Assessment of left atrial appendage function by transesophageal echocardiography. Implic. Dev. Thrombus. Circulation.

[18] Budnik M., Gawałko M., Gorczyca I., Uziębło-Życzkowska B., Krzesiński P., Kochanowski J., Scisło P., Michalska A., Jelonek O., Starzyk K. (2022). Risk of left atrial appendage thrombus in patients with atrial fibrillation and chronic kidney disease. Cardiol. J..

[19] Faroux L., Cruz-González I., Arzamendi D., Freixa X., Nombela-Franco L., Peral V., Caneiro-Queija B., Mangieri A., Trejo-Velasco B., Asmarats L. (2021). Effect of Glomerular Filtration Rates on Outcomes Following Percutaneous Left Atrial Appendage Closure. Am. J. Cardiol..

[20] López-Tejero S., Antúnez-Muiños P., Fraile-Gómez P., Sousa G.B., Rodríguez-Collado J., Herrero-Garibi J., Blanco-Fernández F., Diego-Nieto A., Delgado-Lapeira G.C., Del Villar-Moro M.C.P. (2024). Left atrial appendage occlusion in patients suffering from advanced chronic kidney disease (stage 4 and 5). Long-term follow-up. Catheter. Cardiovasc. Interv..

[21] Osmancik P., Herman D., Neuzil P., Hala P., Taborsky M., Kala P., Poloczek M., Stasek J., Haman L., Branny M. (2020). Left Atrial Appendage Closure Versus Direct Oral Anticoagulants in High-Risk Patients with Atrial Fibrillation. J. Am. Coll. Cardiol..

[22] Freeman J.V., Varosy P., Price M.J., Slotwiner D., Kusumoto F.M., Rammohan C., Kavinsky C.J., Turi Z.G., Akar J., Koutras C. (2020). The NCDR Left Atrial Appendage Occlusion Registry. J. Am. Coll. Cardiol..

[23] Osmancik P., Herman D., Neuzil P., Hala P., Taborsky M., Kala P., Poloczek M., Stasek J., Haman L., Branny M. (2022). 4-Year Outcomes After Left Atrial Appendage Closure Versus Nonwarfarin Oral Anticoagulation for Atrial Fibrillation. J. Am. Coll. Cardiol..

[24] Kefer J., Tzikas A., Freixa X., Shakir S., Gafoor S., Nielsen-Kudsk J.E., Berti S., Santoro G., Aminian A., Landmesser U. (2016). Impact of chronic kidney disease on left atrial appendage occlusion for stroke prevention in patients with atrial fibrillation. Int. J. Cardiol..

[25] Stanifer J.W., Pokorney S.D., Chertow G.M., Hohnloser S.H., Wojdyla D.M., Garonzik S., Byon W., Hijazi Z., Lopes R.D., Alexander J.H. (2020). Apixaban Versus Warfarin in Patients with Atrial Fibrillation and Advanced Chronic Kidney Disease. Circulation.

[26] Genovesi S., Porcu L., Slaviero G., Casu G., Bertoli S., Sagone A., Buskermolen M., Pieruzzi F., Rovaris G., Montoli A. (2021). Outcomes on safety and efficacy of left atrial appendage occlusion in end stage renal disease patients undergoing dialysis. J. Nephrol..

[27] Friberg L., Rosenqvist M., Lip G.Y.H. (2012). Evaluation of risk stratification schemes for ischaemic stroke and bleeding in 182 678 patients with atrial fibrillation: The Swedish Atrial Fibrillation cohort study. Eur. Heart J..

[28] Della Rocca D.G., Magnocavallo M., Van Niekerk C.J., Gilhofer T., Ha G., D’Ambrosio G., Mohanty C.G., Galvin J., Vetta G., Lavalle C. (2023). Prognostic value of chronic kidney disease in patients undergoing left atrial appendage occlusion. Europace.

[29] Chen C., Cao Y., Zheng Y., Dong Y., Ma J., Zhu W., Liu C. (2021). Effect of Rivaroxaban or Apixaban in Atrial Fibrillation Patients with Stage 4–5 Chronic Kidney Disease or on Dialysis. Cardiovasc. Drugs Ther..

[30] Jun M., James M.T., Manns B.J., Quinn R.R., Ravani P., Tonelli M., Perkovic V., Winkelmayer W.C., Ma Z., Hemmelgarn B.R. (2015). The association between kidney function major bleeding in older adults with atrial fibrillation starting warfarin treatment: Population based observational study. Br. Med. J..

[31] Proietti M., Lip G.Y. (2016). Major Outcomes in Atrial Fibrillation Patients with One Risk Factor: Impact of Time in Therapeutic Range Observations from the SPORTIF Trials. Am. J. Med..

[32] Pisters R., Lane D.A., Nieuwlaat R., De Vos C.B., Crijns H.J.G.M., Lip G.Y.H. (2010). A novel user-friendly score (HAS-BLED) to assess 1-year risk of major bleeding in patients with atrial fibrillation: The euro heart survey. Chest.

[33] Hildick-Smith D., Landmesser U., Camm A.J., Diener H.C., Paul V., Schmidt B., Settergren M., Teiger E., Nielsen-Kudsk J.E., Tondo C. (2020). Left atrial appendage occlusion with the Amplatzer™ Amulet™ device: Full results of the prospective global observational study. Eur. Heart J..

[34] Luani B., Genz C., Herold J., Mitrasch A., Mitusch J., Wiemer M., Schmeißer A., Braun-Dullaeus R.C., Rauwolf T. (2019). Cerebrovascular events, bleeding complications and device related thrombi in atrial fibrillation patients with chronic kidney disease and left atrial appendage closure with the WATCHMAN^TM^ device. BMC Cardiovasc. Disord..

[35] Benini Tapias J., Flores-Umanzor E., Cepas-Guillén P.L., Regueiro A., Sanchís L., Broseta J.J., Cases A., Freixa X. (2022). Impacto pronóstico de la enfermedad renal crónica sobre el cierre percutáneo de la orejuela izquierda en la fibrilación auricular: Una experiencia unicéntrica. Nefrología.

[36] Weir M.R., Ashton V., Moore K.T., Shrivastava S., Peterson E.D., Ammann E.M. (2020). Rivaroxaban versus warfarin in patients with nonvalvular atrial fibrillation and stage IV–V chronic kidney disease. Am. Heart J..

[37] Garlo K.G., Steele D.J.R., Nigwekar S.U., Chan K.E. (2019). Demystifying the benefits and harms of anticoagulation for atrial fibrillation in chronic kidney disease. Clin. J. Am. Soc. Nephrol..

[38] Shin J.I., Secora A., Alexander G.C., Inker L.A., Coresh J., Chang A.R., Grams M.E. (2018). Risks and Benefits of Direct Oral Anticoagulants across the Spectrum of GFR among Incident and Prevalent Patients with Atrial Fibrillation. Clin. J. Am. Soc. Nephrol..

[39] Melgaard L., Gorst-Rasmussen A., Lane D.A., Rasmussen L.H., Larsen T.B., Lip G.Y. (2015). Assessment of the CHA2DS2-VASc Score in Predicting Ischemic Stroke, Thromboembolism, and Death in Patients with Heart Failure with and Without Atrial Fibrillation. JAMA.

[40] Abouzid M.R., Kamel I., Saleh A., Vidal Margenat A., Hariharan R. (2024). Assessing Stroke and Mortality Risk in Heart Failure: The CHA2DS2-VASc Score’s Prognostic Value in Patients with and Without Atrial Fibrillation: A Meta-Analysis. Cardiol. Rev..

[41] Lip G.Y. (2011). Implications of the CHA(2)DS(2)-VASc and HAS-BLED Scores for thromboprophylaxis in atrial fibrillation. Am. J. Med..

[42] Levey A.S., Grams M.E., Inker L.A. (2022). Uses of GFR and Albuminuria Level in Acute and Chronic Kidney Disease. N. Engl. J. Med..

