# Supplementary material for: Left Atrial Appendage Occlusion Compared to Anticoagulation in Patients Suffering from Atrial Fibrillation with Advanced Chronic Kidney Disease
Source: J Clin Med. 2025 Aug 12;14(16):5709. doi: 10.3390/jcm14165709 (PMC12386536; doi:10.3390/jcm14165709)

SUPPLEMENTARY APPENDIX

Figure S1. 1-year follow-up Kaplan-Meier plot and estimate for the primary combined endpoint (strok e, AIT, SE, and major bleeding) at 1-year follow-up.

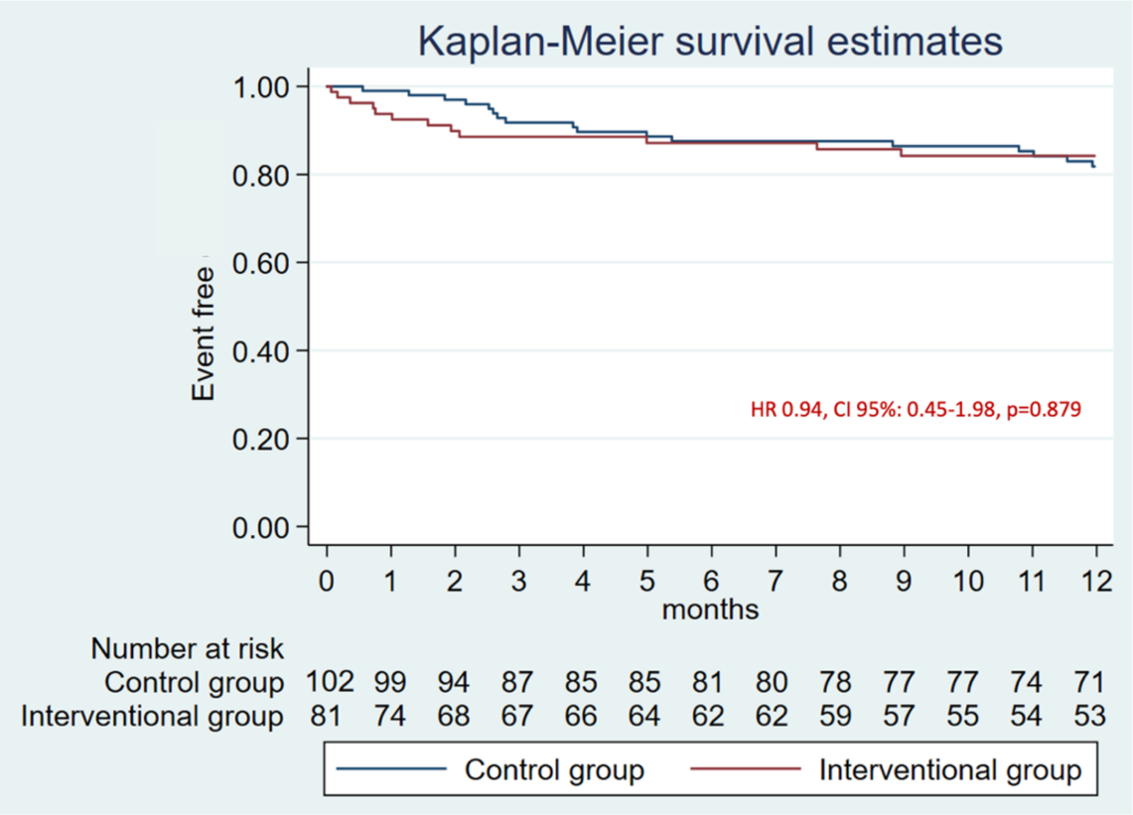

Figure S2. 1-year follow-up Kaplan-Meier plot and estimate for major bleeding.

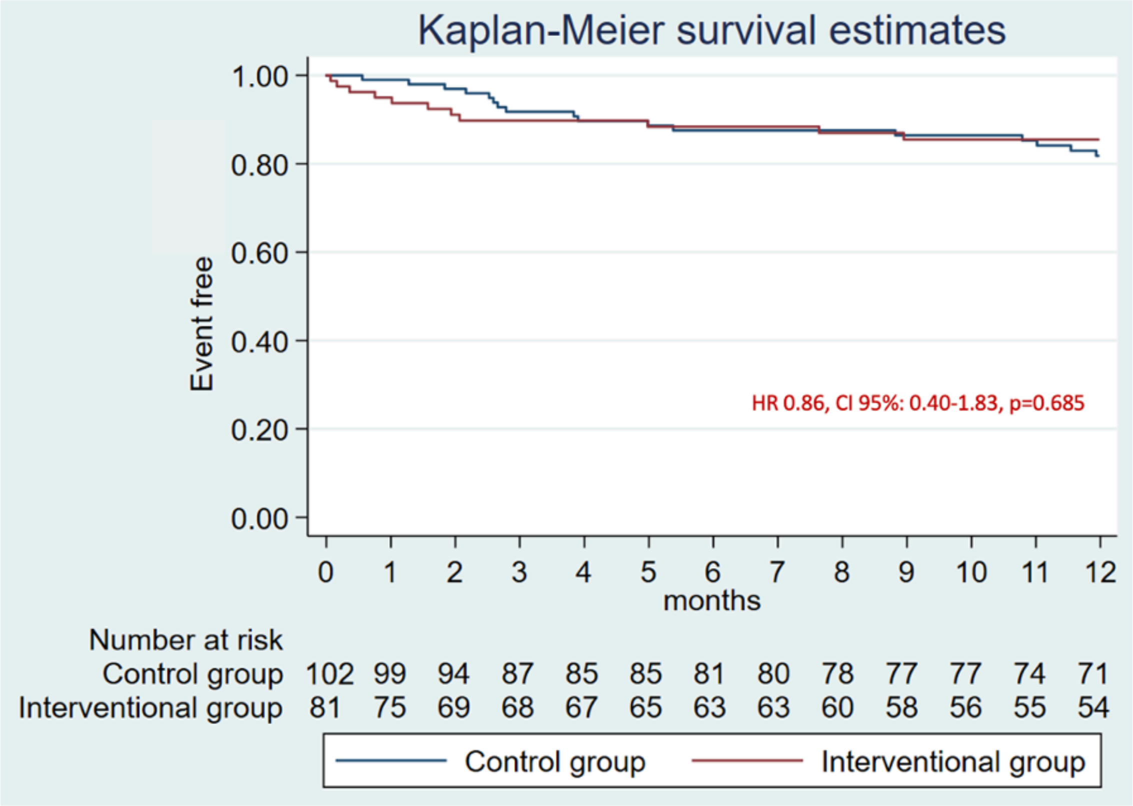

Figure S3. 3-year follow-up Kaplan-Meier plot and estimate for mortality after propensity score matching.

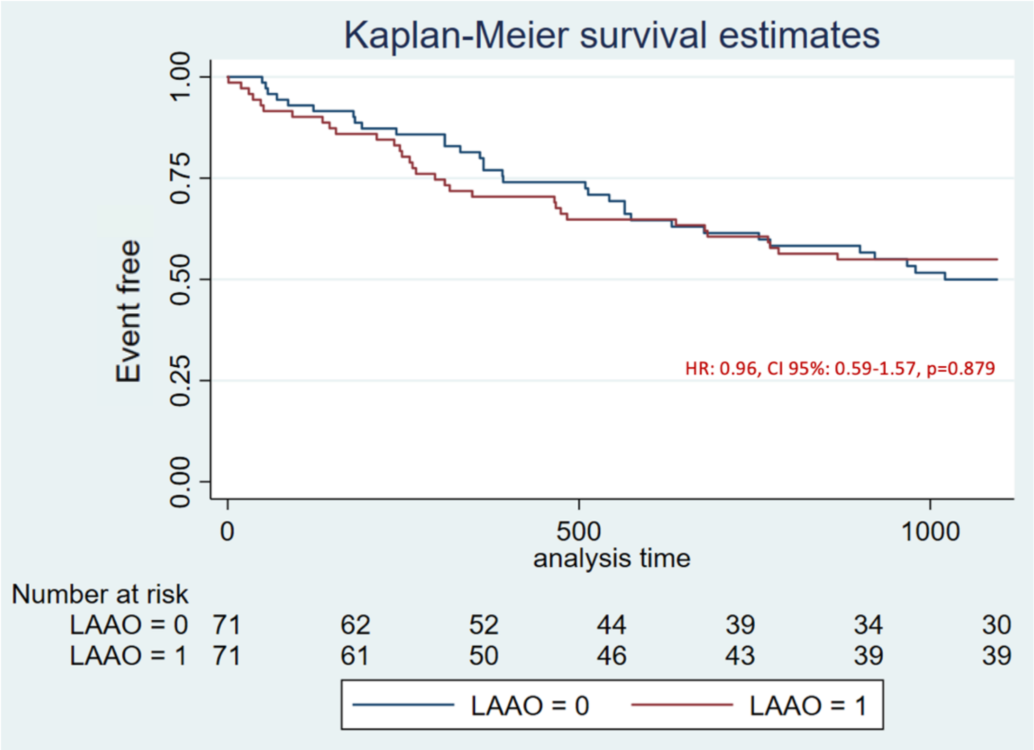

Supplement: Supplementary file 1 [file jcm-14-05709-s001.zip › jcm-3776608-supplementary.pdf]
